# Analysis of Factors Affecting Employment Status of Kidney Transplant Recipients in Selected European Union Member States

**DOI:** 10.3390/ijerph181910284

**Published:** 2021-09-29

**Authors:** Elzbieta Wlodarczyk, Ondřej Viklický, Klemens Budde, Marie Kolářová, Leon Bergfeld, Leszek Paczek, Krzysztof Mucha, Maciej Glyda, Zbigniew Wlodarczyk

**Affiliations:** 1 Department of Geriatrics, Faculty of Medical Sciences, Collegium Medicum in Bydgoszcz, Nicolaus University, 87-100 Torun, Poland; ewlodar@yahoo.com; 2 Institute for Clinical and Experimental Medicine, 140 21 Prague, Czech Republic; onvi@ikem.cz (O.V.); makc@ikem.cz (M.K.); 3 Medizinische Klinik Mit Schwerpunkt Nephrologie und Internistische Intensivmedizin, Charité–Universitätsmedizin Berlin, 10117 Berlin, Germany; klemens.budde@charite.de (K.B.); leon.bergfeld@charite.de (L.B.); 4 Department of Immunology, Transplantology and Internal Medicine, Warsaw Medical University, 02-091 Warsaw, Poland; leszek.paczek@gmail.com (L.P.); kjmucha@gmail.com (K.M.); 5 Department of Transplantology and General Surgery, Poznan District Hospital, 60-479 Poznan, Poland; glydam@wp.pl; 6 Department of Transplantology and General Surgery, Faculty of Medicine, Collegium Medicum in Bydgoszcz, Nicolaus University, 87-100 Torun, Poland

**Keywords:** kidney transplantation, employment, quality of life

## Abstract

Despite an increasing quality of life after renal transplantation, the number of recipients undertaking paid professional work remains relatively low. Employment after kidney transplantation became a new important marker of clinically significant health recovery. Furthermore, for social and economic reasons, returning to work and participation in social life may be considered as an objective parameter that demonstrate the effectiveness of transplantation. The objectives of the following study were to evaluate the factors that determine resuming paid work after renal transplantation, to assess a patient’s decision about returning to professional activity by comparative analysis of renal transplant recipients from Poland, Czech Republic and Germany, and to identify groups of patients exposed to professional exclusion in those EU countries. Five hundred renal transplant recipients from three EU countries were included into the study. The two main research methods used in the study were the SF-36 questionnaire, constructed and validated to assess the quality of life after kidney transplantation and a questionnaire constructed for the purposes of this study. Multifactorial analysis identified several risk factors associated with professional exclusions after kidney transplantation, namely young or advanced age, female gender, lack of education, place of residence in rural areas, long period of illness, and lack of occupational activity before transplantation. Despite the high standards of social care and rehabilitation support, patients in Germany failed to take up professional activity after kidney transplantation in more cases than those in Poland and Czech Republic. Surprisingly, the objective function of the kidney (creatinine level) and the multidimensional assessment of quality of life (SF-36 survey) did not have a significant association with the employment status after renal transplantation.

## 1. Introduction

Transplantation is a method of choice for the treatment of chronic stage V kidney disease and aims not only at extending the life of recipients, but also at improving the quality of life and socio-professional rehabilitation [1]. Returning to work and participation in social life are now objective parameters that demonstrate the effectiveness of transplantation [2,3,4,5]. Excellent results of kidney transplantations may lead to the intuitive assumption that patients after transplantation have the opportunity to return to full or only slightly limited professional activity. In a situation of the enormous financial burden associated not only with transplantation procedure and follow-up treatment itself but also with the costs of the social security system and all other socio-economic costs, it is extremely important to identify and eliminate the factors that may negatively affect patient’s ability or willingness to return to the professional activity.

This study was designed to identify factors determining the effectiveness of occupational rehabilitation in kidney recipients and the reasons for making the decision about returning to the paid work. The study took into consideration the differences in social welfare systems, in terms of legal, political, and economic differences between selected EU countries and how these systems may influence professional rehabilitation.

### Objectives

1. To assess the re-employment rate after kidney transplantation in selected EU member states
2. To identify the factors determining the rate of occupational inactivity after kidney transplantation
3. To evaluate the correlation between the kidney function (as an objective factor) and quality of life (as a subjective factor) and the post-transplant employment status

## 2. Materials and Methods

The study was conducted at five transplant centres, three in Poland (in Poznan, Bydgoszcz, and Warsaw) and the remaining two in Prague and Berlin. The main method used in the study was a diagnostic survey with validated SF-36 Quality of Life Questionnaire assessing the quality of life after kidney transplantation. The SF-36 questionnaire allowed for self-assessment of health and quality of life in various dimensions [6]. A questionnaire containing demographic and clinical variables was constructed for this study and provided information on social and occupational status, demographics and clinical data, parameters concerning the cause of the disease and the subjective assessment of well-being.

One hundred subsequent kidney transplant recipients seen in the outpatient clinic in each centre were approached for the study. The inclusion criteria were: consent for the study, at least 6 months post-transplant, no permanent or supportive dialyses and age between 18 and retirement age (different for each country). 

Questionnaires were handed over to patients during routine visits in the Outpatient Clinics at the transplant centre, according to the following criteria: patients who were recipients of a kidney from a deceased or living donor (regardless of the time from kidney transplantation), stable kidney function (without the need for dialysis, regardless the creatinine level), and legal capacity to take up paid work.

Criteria for the study centres selection were as follows: in Poland—one of the largest and the most active transplant centres, Czech Republic—the similarity of economic development and similar political changes in recent years as the ones in Poland (joining the EU in 2004, together with Poland), Germany, as a reference country—one of the richest countries in the world, with a stable economic situation and a social security system model recognized globally as one of the best health care systems, as well as with an extensive system of vocational rehabilitation [7].

300 questionnaires in Poland, 89 in Germany and 91 in the Czech Republic were returned with a sufficient amount of data for evaluation. The normal distribution of the data was assessed with the Shapiro–Wilk test. Using a two-sided significance test, the demographic data were assessed for a difference between structure indicators. The comparison of continuous distributions was performed using the Mann–Whitney–Wilcoxon test and the Kruskal–Wallis test, together with the multiple comparison test. The relationship between nominal variables was verified using the chi-square independence test or the Fisher’s exact test. Logistic regression was used to analyze the data in order to identify independent factors related to patients after a kidney transplantation surgery taking up/failing to take up work. The Pearson’s correlation coefficient was used to examine the strength of dependence between selected continuous type variables.

## 3. Results

National groups differed in the age distribution (group 30–39 was dominant in Poland), as well as education (secondary education also dominant in Poland vs. Czech Republic and Germany) and place of residence (urban prevailing in Berlin, Germany). The most prominent difference was in the length of pre-transplant dialysis, significantly longer in German patients. Other demographic factors were similar in all three groups (Table 1).

**Figure 1:**
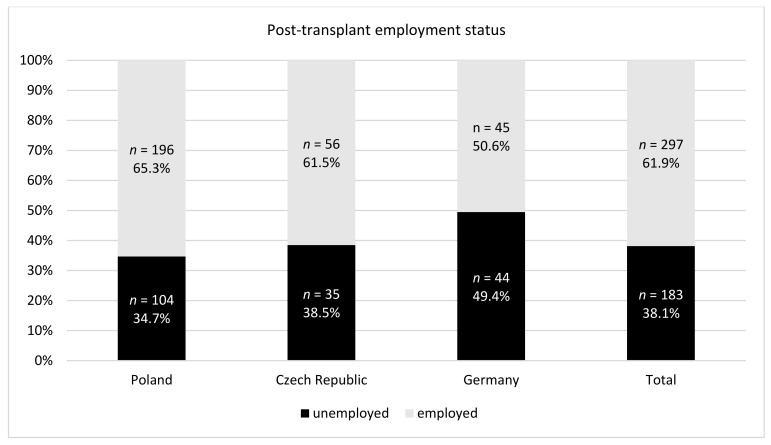
Post-transplant employment status.

In the post-transplant period, most of the recipients either returned to the pre-transplant occupational activity or undertook the employment de novo. However, while in Poland and Czech Republic that proportion was more than 60%, in Germany only 50% of the recipients were professionally active, and the difference between Poland and Germany was statistically significant (Figure 1).

**Figure 2:**
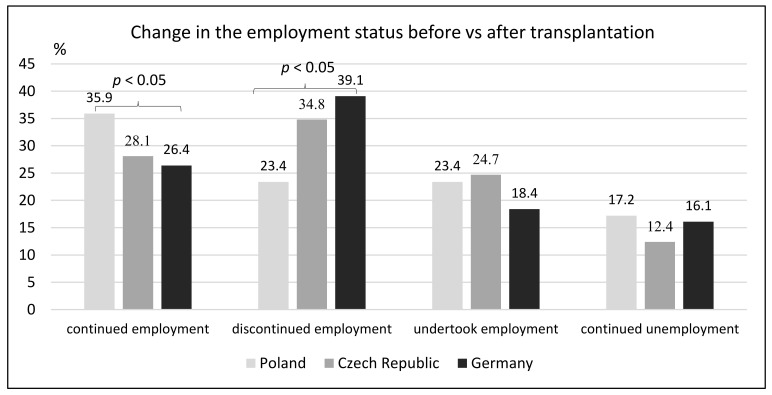
Change in the employment status before vs. after transplantation.

It seems that such difference was caused mostly by the fact (presented below) that a significantly higher proportion of patients in Germany did not continue employment after transplantation (Figure 2)

In order to determine the factors that may contribute to the post-transplant occupational inactivity and subsequent social exclusion, a multifactorial logistic regression analysis was performed (Table 2).

It appeared that such factors as female gender, basic education, younger age, rural place of residency, and unemployment before transplantation increase the risk of post-transplant occupational idleness.

**Figure 3:**
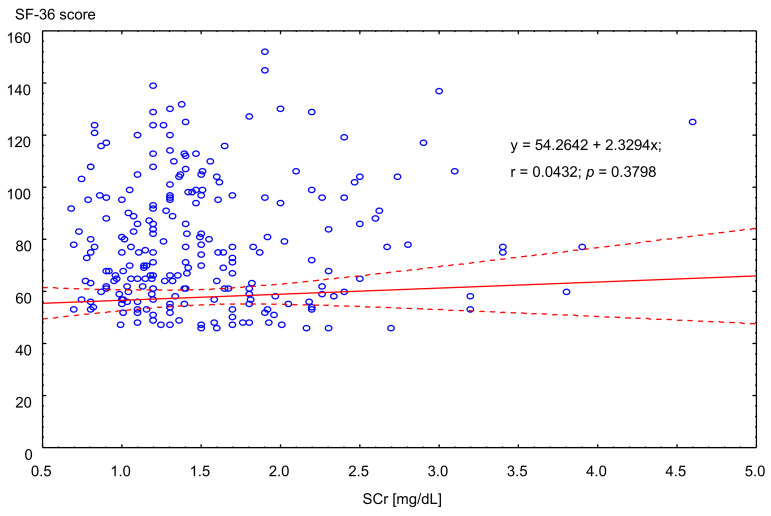
Correlation between serum creatinine level and assessment with SF-36 (combined physical and mental component). Solid line: trend. Dotted lines: ±SD.

We assumed that subjective quality of life assessed with the SF-36 questionnaire would be (at least to some extent) the derivative of objective factors such as kidney function, represented by serum creatinine level. The result was on the contrary: both total and subset of the mental component of quality of life were completely unrelated to the kidney function (Figure 3).

According to the construction of the SF-36, a higher score allocated to the patient means worse mental or physical condition [8]. As expected, unemployed patients in all three national groups presented with lower quality of life than those professionally active. However, patients in Germany, despite the lowest rate of employment, presented with the highest self-estimation of quality of life with the SF-36 (Table 3).

Interestingly, the employment status was not related to kidney function, measured with serum creatinine concentration (Table 4).

## 4. Discussion

Excellent results of kidney transplantation, directed not only at increasing the patient’s survival rate, but also at significant improvements in their quality of life, have been demonstrated already in many studies [9]. It leads to the intuitive assumption that post-transplantation patients also have the opportunity to return to full or only slightly limited professional activities. The resumption of work by renal recipients is extremely important in terms of the social and economic aspects of every country. The present study was designed to identify factors that can determine returning to paid work by kidney recipients, as well as those at risk of professional exclusion. Social welfare systems, their forms and significant differences between selected EU countries and political changes in this aspect were also analyzed for their impact on patients’ decisions.

The most surprising and unexpected result of this study was the significantly smaller percentage of German kidney recipients returning to work, as compared to Poles and Czechs. It would seem that the German extensive social welfare system, recognized as the best and showcase system in the world, encourages people to take up a job [10]. The results of the study show that it can have quite the opposite effect. A significantly lower percentage of resumed paid work among German patients, especially those with higher education, can be explained by very liberal principles of convalescence and comprehensive rehabilitation or fringe benefit (sick leave of 546 days with the possibility of extending this period, high sickness benefits, extensive additional financial and rehabilitation support) [10,11]. On the other hand, the disability pension in Poland (2018) was relatively low (between 200 and 500 EUR/month). Although Polish kidney recipients receiving disability pension may lose coverage eligibility if they undertake employment and have increased personal revenue, the much higher income from employment may determine the decision. This seems to be the case with some insurers in the USA as well and a survey conducted in 2014 in the USA came to similar conclusions. Employment in the US is treated as an important indicator of clinically significant recovery. The researchers tried to answer the question why, despite the high growth in the quality of life, the employment level is very low and is not growing in comparison to the general population. Over 100,000 patients from the UNOS database (United Network for Organ Sharing) were analyzed. The authors of that study indicated that finances and the issuing of insurance by private companies (not by state insurers) were strong motivating factors behind taking up professional activities [2].

Over half of the respondents in our study were patients in the 50–65 age group. The demographic ageing of societies also influenced the principles of transplantology, such as raising age of donors and recipients (the proportion of older recipients in the Polish group doubled over the last ten years). Advanced age significantly affects the professional status because of the adaptation difficulties at work, possible need for retraining, the temptation to remain on a pension until retirement, and the reluctance of employers to employ people of the pre-retirement age. Similar results were obtained by other researchers [2,12,13,14,15,16,17]. Another group at risk of professional exclusion (Germany, Poland) contains young people aged 20–29, whose illness hindered their chance for education.

Patients with secondary and higher education have a higher probability to take up work after kidney transplantation. The key aspect seems to be the attitude of well-educated people towards work perceived as a value, as well as the capacity and physical capabilities of the recipients and a much larger selection of job vacancies with the prospect of good remuneration. Similar conclusions were included in a Swiss cohort study (689 patients) [12].

The pre-transplantation professional status seems to be another important factor impacting post-transplantation employment. It is significantly more probable to return to or take up work after transplantation for people who had worked in the period of illness and dialysis. Similar conclusions were drawn from the analysis by Tzvetanov, D’Amico, and Walczak et al. [2]. In addition, active professional status, as a predictor of returning to work after transplantation, had already been identified and confirmed in many analyses [12,13,14,15]. Unfortunately, it seems that this factor is not taken into consideration by decision-makers or authorities responsible for occupational rehabilitation, prevention of professional exclusion, as well as the social security system [3,13,15,16,17,18].

In the case of another variable, gender, there are several possible interpretations of the results. The significantly smaller rate of women returning to work may be caused by the fact of professional discrimination on the grounds of sex (confirmed in other studies) but also due to traditional social roles attributed to women, such as the role of a mother and housewife. This attitude seems to be more prominent in the so-called “new EU members” [4,19].

Surprisingly, there was no correlation between the graft function (determined by a serum creatinine level) and professional status. At the same time, it was demonstrated that the working patients considered their quality of life and their health to be significantly better than non-working patients. This leads to the conclusions analogous to the study by Canadian scientists who reported that respondents who had not started work after transplantation assessed their well-being and overall health at a lower level [13]. Furthermore, in another retrospective study, US researchers concluded that active occupational status before and after transplantation had a beneficial effect on reducing the risk of losing a transplanted kidney and reducing the mortality of kidney recipients [3].

Perhaps not only the psychological aspect of positive self-esteem but also comprehensive information about the possibilities and privileges may influence the re-employment rate. Among the national groups included in our study, Polish patients had the most comprehensive information regarding their health status and work possibility, which correlated positively with the re-employment rate. Similar conclusions were drawn from a study from Belgium [4]. The results of the study conducted by the Dallas Transplant Institute in the USA (kidney recipients 18–55 years of age) are also worth mentioning. As part of the Job Club Vocational Rehabilitation (intervention model) Program, patients were involved in psychoeducation, trained in terms of recognizing individual problems and using forms of support, as well as had developed self-motivation and employment-seeking skills. These patients not only evaluated their health at a good level in all its aspects (measured using the SF-36 questionnaire) but also took up professional work more frequently than others [4,5,18].

## 5. Conclusions

1. Gender, low level of education, and rural residency correlates with post-transplant occupational inactivity
2. Unemployed kidney recipients present with lower quality of life, but it is not linked with kidney function
3. Superior post-transplant social provisions may create the conditions for occupational idleness.

## Figures and Tables

**Table 1:** Demographic data.

	Poland	Czech Republic	Germany	*p*
Female	*N* = 176 (59.5%)	*N* = 59 (64.8%)	*N* = 45 (50.6%)	ns
Male	*N* = 120 (40.55%)	*N* = 32 (35.2%)	*N* = 44 (49.4%)	ns
Age (years)				
20–20	*N* = 22 (7.4%)	*N* = 5 (5.5%)	*N* = 10 (11.2%)	ns
30–39	*N* = 68 (23.0%)	*N* = 9 (9.9%)	*N* = 8 (9.0%)	0.001
40–49	*N* = 75 (25.3%)	*N* = 19 (20.9%)	*N* = 24 (27.0%)	ns
50–65	*N* = 131 (44.3%)	*N* = 58 (63.7%)	*N* = 46 (51.7%)	ns
Education				
Basic	*N* = 19 (6.4%)	*N* = 7 (7.7%)	*N* = 8 (9.0%)	ns
Professional	*N* = 82 (27.7%)	*N* = 37 (40.7%)	*N* = 41 (46.1%)	0.0424
Secondary	*N* = 127 (42.9%)	*N* = 34 (37.4%)	*N* = 21 (23.6%)	0.011
University	*N* = 68 (23.0%)	*N* = 13 (14.3%)	*N* = 19 (21.4%)	ns
Place of residency				
Rural	*N* = 112 (37.8%)	*N* = 31 (34.1%)	*N* = 22 (24.7%)	ns
Suburban	*N* = 110 (37.5%)	*N* = 36 (39.6%)	*N* = 16 (18.0%)	ns
Urban	*N* = 73 (24.7%)	*N* = 24 (26.4%)	*N* = 51 (57.3%)	0.002
Time of pre-transplant dialysis (months: mean, SD)	27 (±23)	28 (±25)	59 (±45)	0.000
Time since transplantation (months: mean, SD)	94 (±66)	90 (±76)	89 (±65)	0.777

**Table 2:** Multifactorial logistic regression analysis of factors influencing post-transplant employment.

	OR (95% CI)	*p*
Gender M vs. F	1.9 (1.31–2.75)	0.0007
Age (years)		
30–39 vs. 20–30	2.69 (1.21–5.98)	0.0156
40–49 vs. 20–30	2.85 (1.32–6.12)	0.0074
50–65 vs. 20–30	0.94 (0.47–1.91)	0.8739
Education		
professional vs. basic	2.92 (1.2–7.11)	0.0179
Secondary vs. basic	5.89 (2.44–14.25)	0.0001
University vs. basic	9.78 (3.82–25.02)	0.0000
Place of residency		
Suburban vs. rural	1.93 (1.24–2.99)	0.0034
Urban vs. rural	1.89 (1.21–2.97)	0.0054
Employment during pre-transplant period of dialyses (yes vs. no)	1.68 (1.16–2.43)	0.0060

**Table 3:** SF-36 score in the unemployed vs. employed group.

	Unemployed		Employed		*p*
	*n*	SF36 Median (Q1–Q3)	*n*	SF36 Median (Q1–Q3)	
Poland	103	74 (47–99)	185	53 (30–69)	<0.01
Czech Republic	32	70.5 (47–92)	53	44.5 (24–57.5)	<0.05
Germany	42	59.5 (38.5–73.5)	41	37.5 (22–50)	<0.01
All patients	177	69.9 (45.4–81.5)	279	49.1 (25.2–58.6)	<0.05

**Table 4:** Serum creatinine concentration in the unemployed vs. employed group.

	Unemployed		Employed		*p*
	*n*	Average SCr (mg/dL)	*n*	Average SCr (mg/dL)	
Poland	103	1.61 (0.55–4.60)	185	1.52 (0.73–3.90)	ns
Czech Republic	32	1.68 (0.59–3.05)	53	1.54 (0.68–4.64)	ns
Germany	42	1.78 (0.90–3.40)	41	1.64 (0.86–3.20)	ns
All patients	177	1.66	279	1.54	ns

## Data Availability

The data presented in this study are available on request from the corresponding author.
